# Meditation Practice Improves Short-Term Changes in Heart Rate Variability

**DOI:** 10.3390/ijerph17062128

**Published:** 2020-03-23

**Authors:** Kang-Ming Chang, Miao-Tien Wu Chueh, Yi-Jung Lai

**Affiliations:** 1 Department of Photonics and Communication Engineering, Asia University, 41354 Taichung, Taiwan; changkm@asia.edu.tw; 2 Department of Medical Research, China Medical University Hospital, China Medical University, 40402 Taichung, Taiwan; 3 Sacred Light Heart Chan Association, 103613 Taipei, Taiwan; miaotein@buddhachan.org; 4 Department of Early Childhood Educare, Wu Feng University, 62153 Chiayi, Taiwan

**Keywords:** heart rate, heart rate variability, meditation, blessing

## Abstract

Background: It is well known that meditation improves the physical and psychological condition of its practitioners. This study investigated the heart rate variability response of meditation practitioners in two Chan master teaching environments, namely face-to-face and video classes. Methods: Experimental sessions were conducted, one featuring face-to-face classes and the other featuring video classes. The difference in participants’ physiological parameters (blood pressure and heart rate variability) between the two experimental sessions was determined. In the first session, physiological parameters were recorded twice, before and after one teaching course, and the second session took place one month after the first. The first and second sessions had 45 and 27 participants, respectively. Those involved in the first experiment had no experience with meditation, whereas participants in the second experiment had practiced meditation for an average of 9 years (range = 1 to 27 years). Both experiments were conducted once a week, with each session lasting 1.5 h. Results: For both experiments, both heart rate and heart rate variability by age significantly decreased after one teaching course. Conclusions: Chan meditation practitioners benefit from receiving both face-to-face and video class teaching from a Chan master.

## 1. Introduction

Meditation benefits both the physiology and psychology of its practitioners. It enables them to feel joy, to be relaxed, and to be in control of their emotions, especially anger [1]. Meditation also improves its practitioners’ concentration and physical immunity, enabling them to feel more energetic [2]. The physiological parameters related to these feelings can mostly be attributed to the activity of the autonomic nervous system; which is divided into the sympathetic nerves and parasympathetic nerves. The sympathetic nerves accelerate the heart rate (HR) and enhance a person’s perception of their physical strength, whereas the parasympathetic nerves slow the HR and make a person feel relaxed and ready for sleep. Frequency domain parameters are common physiological parameters related to the autonomic nervous system, and they are distinguished as low frequency (LF) and high frequency (HF) types. LF parameters are associated with the mixed activity of the sympathetic and parasympathetic nerves, whereas HF parameters are associated with the activity of the parasympathetic nerves [3]. Studies have discovered that different meditation techniques evoke different autonomic nervous responses. These studies are listed in Table 1. 

Heart Chan meditation is a meditation method that stems from the Chan school of Buddhism. It has been taught, mainly in Taiwan, by the Chan master Wu Chueh Miao-Tien, who is the eighty-fifth Patriarch Chan master. Currently, Heart Chan meditation has more than 100,000 practitioners [11]. Heart Chan meditation encompasses four dimensions of the physical, conscious, subconscious, and the spirit and wisdom of the universe. Through the practice of breathing and concentration, the observation of chakra points within the body, and being in sync with the teaching of the Chan master to enter the state of Chan, practitioners can surpass their physical, mental, and subconscious states and enter into the spiritual realm [12]. The synchronization between the Chan master and practitioners can be either face-to-face or through video classes. Many studies have discussed the benefits of Heart Chan meditation on physical and mental health as well as its practical principles [13]. These studies have mostly analyzed Heart Chan meditation with respect to its resultant physiological signals, finding that this meditation approach improves happiness [14], anxiety [15], the regulation of heartbeat and breath [16], brain harmony [17], in addition to enabling practitioners to perceive an inner light [18]. Most Chan practitioners learn meditation through video classes; they gather in a meditation center at regular times to watch teaching videos. Only a few practitioners attend meditation classes that are taught by a Chan master in person. The practitioners have reported such face-to-face classes as enabling them to more easily comprehend the benefits of practicing meditation, such as feeling more energetic, becoming healthier, and being in a pleasant mood. However, the differences between face-to-face and video classes have not been deeply investigated.

Although numerous studies have discussed meditation-induced heart rate variability (HRV) changes, the present study attempted to investigate the relationship between biological HRV age, and the practice of Heart Chan meditation. HRV was noted to decrease with age, and a preliminary reference norm for such a relationship is available [19]. Practitioners of Heart Chan meditation tend to feel younger and more vigorous after practicing. However, these perceptions are all short-term responses to meditation, and this study aimed to determine whether these feelings are related to objective physiological signals. In the context of the contemporary trend toward body care, whether Heart Chan meditation practice can indeed help practitioners feel younger and improve their health is a worthy topic of research.

## 2. Materials and Methods

### 2.1. Participants and Experimental Design

This study comprised two experiments: one where a face-to-face class was conducted and the other where a video class was conducted. All participants were briefed on the experimental procedure before they agreed to participate, where they gave their written informed consent by signing an experimental consent form. The experiment was approved by the Medical Research Ethics Committee of Asia University (number 10506007). Participants’ information and the experimental procedures are listed in Table 2.

Experiment 1 was conducted as follows. The Chan master taught meditation in face-to-face classes, and the learners had no experience with meditation. This experiment was divided into two subexperiments. In subexperiment 1A, the participants’ physiological parameters before and after the first face-to-face meditation session were measured to determine the change in parameters. In total, the data of 22 participants were recorded, but only 18 participants were successfully enrolled after the exclusion criteria was applied. Their ages ranged from 24 to 71 years, with the mean being 47.9 years (standard deviation; SD = 17.2 years), and there were 12 men and 6 women. Subexperiment 1B involved participants in subexperiment 1A and additional recruited participants. Physiological parameters were observed over 1 month. In total, the data of 50 participants were recorded, among whom 45 participants were enrolled after the exclusion criteria were applied. Their ages ranged from 25 to 68 years, with a mean of 46.7 years (SD = 12.7 years), and there were 17 men and 28 women.

Experiment 2 involved video classes. The participants were regular practitioners of Heart Chan meditation who practiced at the venue of the experiment. They were asked to attend the experiment’s class every week at a designated time point, and their data were collected. The changes in their physiological parameters during the video-based meditation learning were recorded. Their physiological parameters were measured before and after each weekly video class over 4 weeks. Four measurements for each participant were taken to ensure consistency, and the data of 51 participants were recorded. However, participants who did not complete the experiment and who exhibited more than 10 instances of irregular heartbeat were excluded. In total, 27 participants were successfully enrolled (age range, 20 to 68 years; mean age = 49.2 years, SD = 14.7 years; men, *n* = 5, women, *n* = 22). Participants had practiced Heart Chan meditation for an average of 9 years (range = 1 to 27 years).

Both face-to-face and video classes were taught with the same teaching method. The duration of each session was 90 min. The Chan master detailed the principles of and precautions to be taken when practicing meditation and guided the participants when practicing meditation together. All participants sat on the floor in a cross-legged position and practiced breathing and concentrating on the chakra points within their bodies. Synchronization between the minds of the Chan master and practitioners is a crucial factor for entering a deeper meditative state.

For the experiments, the participants were asked to rest for 20 min before measurement. They were asked to sign an informed consent form after the researcher briefed them on the conduct of the experiment. Their physiological parameters were measured using ANSWatch® wrist monitors (Taiwan Scientific Corp., New Taipei City, Taiwan) and the duration of the measurement was 7 min. Values for the participants’ physiological parameters were shown on the device, and the data were output to Excel after the experiments, for statistical analysis.

### 2.2. Cardiovascular Parameters

**Figure 1:**
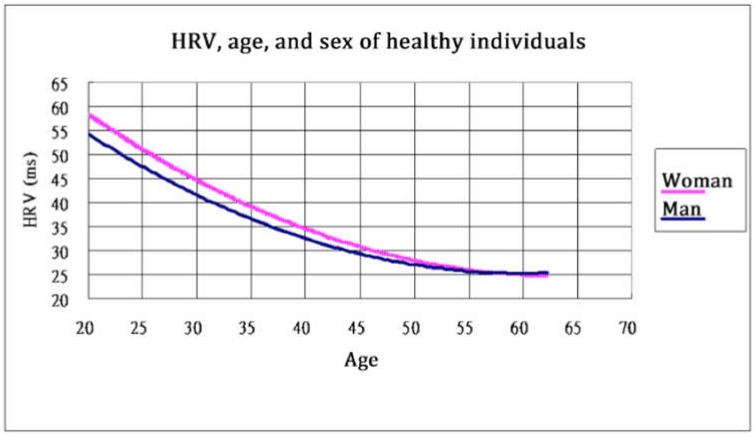
The relationship between heart rate variability and real age (data originally from [19], Figure 3).

Table 3 lists the physiological parameters analyzed in this study. These parameters were automatically produced by the ANSWatch® wrist monitors [19]. The normal values of each autonomic nervous system activity, according to ANSWatch, are shown in Figure 1.

### 2.3. Statistics

#### 2.3.1. Descriptive Statistics

Measurements for each physiological parameter in each experiment were taken at a total of four time points. For experiment 1, the time points were before and after the first session and during the midterm pre- and post-test. For experiment 2, the time points were before and after the first session and during the pre- and post-test 1 month after the first session of experiment 2. Mean values and standard deviations of the measurement data were calculated.

#### 2.3.2. Paired t-test

1. For the first session of subexperiment 1A, paired t-tests were conducted to compare measurements obtained before the first session and after the session; these two sets of measurement data were called (1A)_Pre and (1A)_Post, respectively.
2. For subexperiment 1B, paired t-tests were conducted to compare measurements obtained before the first session with measurements obtained after 1 month of practice; these two sets of measurement data were called (1B)_Pre and (1B)_Post, respectively.
3. For the first session of subexperiment 2A, paired t-tests were conducted to compare measurements obtained before the first session with measurements obtained after the session’s training course; these two sets of data were called (2A)_Pre and (2A)_Post, respectively.
4. For the training course conducted after 1 month in subexperiment 2B, paired t-tests were conducted to compare measurements obtained before the first session with measurements obtained after the course; these two sets of data were called (2B)_Pre and (2B)_Post, respectively.
5. The difference between the biological HRV age and real age for the aforementioned eight measurements; specifically between (1A)_Pre, (1A)_Post, (1B)_Pre, and (1B)_Post as well as between (2A)_Pre and (2A)_Post, and between (2B)_Pre and (2B)_Post, were examined by performing paired t-tests. The significance level was 0.05.

## 3. Results

The results of the two experiments are summarized in Table 4 and Table 5.

According to Table 4, SYS, DIA, and pulse pressure did not exhibit significant changes before or after the first session or after 1 month of meditation learning. The average HR decreased after the first session from 76 to 70 (*p* = 0.02 *), and the HRV age decreased from 46.1 to 33.6 (*p* = 0.039 *). The participants’ change in HRV age from before to after the first session was examined. Among the four participants aged 60 years or older in the pre-test, three participants exhibited a significantly decreased HRV age. Furthermore, among participants whose average age was 30 years, the three participants who were younger than 30 years had an average HRV age of 45 years in the pre-test (i.e., they had presenility), and two participants exhibited a reduced HRV age to an average of 24 years. As for the autonomic nervous system, the mean values of LF and HF power increased only during the 1-month follow-up period, whereas LF% did not change. This indicated that after 1 month of meditation practice, the activity of the autonomic nervous system increased, enabling the participants to be more energetic. The increase in HF power was particularly substantial (*p* = 0.013 *), indicating that the participants felt relatively relaxed. In addition, after 1 month of practice, their HRV age still decreased in comparison with their real age, which implies that this decrease can continue for a long time.

As presented in Table 5, the results of experiment 2 exhibited similar trends as those of experiment 1. First, in both experiments, the after-session HR was lower than the before-session HR. Second, the HRV age decreased by 6–8 years after the session, whereas the HRV increased. The differences between the results of experiments 1 and 2 were that SYS slightly increased after the first session (from 120 to 124, *p* = 0.03 *), and LF% significantly decreased following the session conducted 1 month after the first session (from 51.9 to 41.1, *p* = 0.038 *).

## 4. Discussion

This study furnished several noteworthy findings. First, this study used biological HRV age to represent the short-term change resulting from Heart Chan meditation practice and found that the participants’ HRV age and HR both decreased. The decrease of HRV age was caused by the increase in HRV, which denotes the increase in the total power of the autonomic nervous system’s activity. As illustrated in Figure 1, HRV decreased as age increased. By contrast, if only HRV is used for analysis (with HRV age excluded), the difference may not be significant; significant differences were noted after the conversion of HRV into HRV age, especially in middle-aged and older adults, because the HRV of such adults is low. If only the absolute value of HRV is used for comparison, statistically significant differences cannot be easily determined. However, if the HRV value is converted into real age, the difference can be substantial. A slight HRV increase can reflect a substantial change in age. Therefore, HRV age can be an effective parameter for testing the physiological changes in middle-aged and older adults who practice meditation. Furthermore, this study determined that HR significantly decreased with increases in HRV. This implies that Heart Chan meditation can enhance autonomic nervous system activity without imposing a burden on the heart. Compared with other activities such as regular exercise, which cause both HRV and HR to increase, Heart Chan meditation can lead to decreased HR; this is associated with an increase in HF activity. Therefore, Heart Chan meditation is suitable for people who cannot perform intensive exercise because of poor cardiovascular conditions.

Differing from findings from other studies on meditation, as well as the results for HR and HRV age in this study, findings for the LF and HF parameters indicated no significant differences. In both experiments, LF% decreased, and HF% increased after the first session, albeit nonsignificantly so. However, the results of the 1-month follow-up also revealed an increase in HF%; this may be associated with the practice method of Heart Chan meditation. In the two experiments of this study, data were measured before and after sessions with 2 h between measurements, during which the participants sat in the venue practicing Heart Chan meditation. The practice method includes concentration, adjusted breathing, and perceiving inner peace, and advanced practitioners can resonate with the energy of the Chan master’s blessings. These activities are nonintensive, and practitioners are required to keep their bodies still [14]. Therefore, LF% and HF% did not change significantly after 2 h. However, after 1 month, HF% increased significantly, which is consistent with the results of a previous study [16]; this indicates that Heart Chan meditation may be effective for improving HF% in the long term.

This study compared the practitioners participating in face-to-face sessions with those participating in video meditation classes, with respect to their physiological parameters. The participants comprised of beginners and experienced practitioners. The results revealed that the beginners’ HRV improved after 1 month, indicating that the meditation class made them feel rejuvenated. By contrast, experienced practitioners exhibited different HRV values before and after one session, but their HRV values did not change after 1 month. One possible explanation may be that the experienced practitioners had improved their physiological condition to the point where they could improve it no further, and thus did not exhibit further improvement during the study period; however, each meditation session was still helpful for them. This explanation requires further examination. In addition, this study noted that video and face-to-face learning exerted the same effect. The contribution of this study is its measurement of data at the meditation venue and the face-to-face classes rather than at university laboratories, as other studies have done. Heart Chan meditation is characterized by the teaching of meditation through synchronization between the Chan master and practitioners, which is similar to the giving of blessings. Although face-to-face blessing is the most direct, the meditation center is also a place being blessed. Hence, video learning in the meditation center is as effective as face-to-face learning.

In this study, because few face-to-face classes were available, most data were from beginners in meditation. Data remain scarce from beginners participating in video classes and experienced practitioners participating in face-to-face classes. We will attempt to collect such data in future research for comparison. Additionally, the experiments in this study were conducted in the same meditation center. Further research is required to determine whether a study conducted in university laboratories will yield different results.

## 5. Conclusions

This study discussed the changes in physiological parameters of Heart Chan meditation practitioners and found that a short-term class could lower participants’ HR and their HRV age. The HRV age was adopted, leading to the understanding that the short-term class could result in a decreased HRV age for participants. In addition, both the face-to-face and video classes were effective at improving the activity of the autonomic nervous system.

## Figures and Tables

**Table 1:** Previous meditation studies involving autonomic nervous responses.

Ref.	Meditation Name	Finding
[4]	Hindu tantric meditation	Increased HR
[5]	Deity (Vajrayana tantric)	Decreased HF
[5]	Vipassana	Increased HF and decreased LF/HF
[5]	Shamatha (Kasina visualization)	Decreased LF/HF
[6]	Zen concentrative meditation	Increased HF and decreased LF/HF
[7]	Concentration meditation: focuses on the breath to achieve the *Samadhi* state	R–R time series signal tends to shift toward a specific frequency to form a resonant peak
[8]	Inward-attention meditation	Decreases in LF/HF ratio and LF norm; increase in HF norm
[6]	Zen concentrative meditation	Increased HF and decreased LF/HF
[9]	Intensive vipassana meditation	Increased HF
[5]	Deity (Vajrayana tantric)	Decreased HF
[5]	Vipassana	Increased HF and decreased LF/HF
[5]	Shamatha (Kasina visualization)	Decreased LF/HF
[10]	Yoga	Increased HF; decreased LF; decreased LF/HF decrease

**Table 2:** Experiment design information.

Experiment Information	Experiment 1: Face-To-Face Classes		Experiment 2: Video Classes	
Time points where subexperiments were conducted	(1A) After the first class	(1B) After one month	(2A) Between classes	(2B) One month later, between classes
Participant profile	Volunteers; no experience with meditation.		Regularly practiced Heart Chan meditation for years.	
Exclusion criteria	Participants who did not complete the experiment and those who exhibited more than 10 instances of irregular heartbeat were excluded.			
Numbers of participants (included/all)	18/22; 12M/6F	45/50; 17M/28	27/51; 5M/22F	
Participant ages (minimum, maximum); (mean, standard deviation)	(24,71); (47.9; 17.2)	(25,68); (46.7; 12.7)	(20,68); (49.2; 14.7)	
Conduct of the class	The duration was 90 min. Participants sat on the floor in a cross-legged position and practiced breathing and concentrating on chakra points within the body, as well as synchronizing with the teaching of the Chan master to enter the state of Chan.			
Experience with meditation	No meditation experience		Average 9 years (minimum 1 year; maximum 27 years)	
Survey and measurement	Consent and personal information survey; physiological measurement by ANSWatch® wrist monitors.			

**Table 3:** Definitions of the physiological parameters and their physiological relevance.

Name	Definitions and Physiological Relevance
Systolic blood pressure (SYS)	The blood pressure when the heart contracts to pump blood to the body, with a normal value ranging between 90 and 140.
Diastolic blood pressure (DIA)	The blood pressure when the heart rests between beats, with a normal value ranging between 50 and 90.
Pulse pressure	The gap between SYS and DIA, with a normal value being 30–50 mmHg.
Number of heartbeats per minute (HR)	The average number of times the heart beats per minute within 5 min.
Number of occurrences of arrhythmia	The number of occurrences of arrhythmia within 5 min. If the value is >20, participants should undergo a physical examination to understand the causes of the arrhythmia.
Activity of the autonomic nervous system (HRV)	The variation in the time interval between heartbeats.
LF power of the autonomic nervous system (LF)	Spectral power of the LF R–R interval (0.04–0.15 Hz), which is related to the activity of the sympathetic nerves.
HF power of the autonomic nervous system (HF)	Spectral power of the HF R–R interval (0.15–0.4 Hz), which is related to the activity of the sympathetic nerves.
LF power ratio of the autonomic nervous system (LF%)	Spectral power ratio of the LF R–R interval. LF% = LF / (LF + HF). The normal range is 30–70%.
Age of the autonomic nervous system activity (HRV age)	The age that the HRV data norm corresponds to. HRV decreases as age increases ([19], Figure 1).

**Table 4:** Blood pressure waves obtained from experiment 1.

Blood Pressure Parameters	(1A)_Pre	(1A)_Post	*p*-Value	(1B)_Pre	(1B)_Post	*p*-Value
SYS	113.8 (13.6)	115.7 (17.4)	0.38	121.2 (18.7)	120.2 (16.8)	0.66
DIA	77.1 (9.1)	78.1 (10.7)	0.63	82.3 (11.2)	81.8 (10.9)	0.70
SYS-DIA	36.6 (5.4)	37.6 (8.5)	0.56	38.9 (9.9)	38.4 (8.8)	0.76
HR	76 (6.3)	70.3 (5.7)	0.02 *	74.3 (13.7)	73.5 (13.8)	0.62
HRV	33.6 (16.0)	44.2 (19.0)	0.058	37.1 (15.3)	44.8 (20.6)	0.014
HRV age	46.1 (16.8)	33.6 (13.8) ^a^	0.039 *	42.2 (20.2)	36.8 (18.6) ^b^	0.07
LF	684.1 (1568.1)	677.9 (1229.2)	0.96	440.9 (711.9)	708.5 (987.5)	0.10
HF	173.6 (102.8)	334.8 (405.6)	0.17	297.2 (257.2)	494.8 (580.9)	0.013 *
LF %	60.6 (18.4)	55.6 (20.2)	0.2	53.2 (19.8)	53.2 (24.8)	0.99

Notes: All data are represented in terms of mean and standard deviation (in parentheses). α = 0.05; ^a^ *p* = 0.02; ^b^ *p* = 0.002; p values marked by with a superscripted a and b are from paired t-test results of the difference between heart rate variability (HRV) age and real age. * *p* <0.05.

**Table 5:** Blood pressure waves obtained from experiment 2.

Blood Pressure Parameters	(2A)_Pre	(2A)_Post	*p*-Value	(2B)_Pre	(2B)_Post	*p*-Value
SYS	120 (17)	124 (21)	0.03 *	117 (21)	118 (19)	0.35
DIA	80 (10)	80 (11)	0.48	77 (11)	78 (10)	0.10
SYS-DIA	40.1 (11.5)	43.7 (13.9)	0.022 *	40.5 (14.0)	40.2 (11.8)	0.8
HR	78 (10)	73 (8)	<0.0001 ***	80 (11)	73 (10)	<0.001 ***
HRV	35 (19)	39 (19)	0.015 *	32 (15)	37 (17)	0.013 *
HRV age	47.5 (19.8)	41.5 (17.7) ^c^	0.009 **	50.6 (21.9)	42.7 (14.7)	0.019 *
LF	440.1 (906.2)	403.7 (691.8)	0.36	387.2 (702.4)	242.3 (308.3)	0.16
HF	443.1 (944.5)	398.0 (513.4)	0.34	271.8 (349.4)	373.2 (395.1)	0.054
LF %	50.6 (24.6)	46.4 (24.5)	0.27	51.9 (27.2)	41.1 (22.6)	0.038 *

Notes: all data are represented in terms of mean and standard deviation (in parentheses). α = 0.05; ^c^ *p* = 0.04, from the paired *t* test results for the difference between heart rate variability (HRV) age and real age. * *p* < 0.05; ** *p* < 0.01; *** *p* < 0.001.
